# Differential expression of *p42.3* in low- and high-grade gliomas

**DOI:** 10.1186/1477-7819-12-185

**Published:** 2014-06-14

**Authors:** Weiqing Wan, Xiaoqing Xu, Guijun Jia, Wenmei Li, Junmei Wang, Tong Ren, Zhen Wu, Junting Zhang, Liwei Zhang, Youyong Lu

**Affiliations:** 1 Department of Neurosurgery, Beijing Tiantan Hospital Capital Medical University, No.6 Tiantan Xili, Dongcheng District, 100050 Beijing, People’s Republic of China; 2 Key Laboratory of Carcinogenesis and Translational Research (Ministry of Education), Peking University Cancer Hospital/Institute, No. 52 Fucheng Road, Haidian District, 100142 Beijing, People’s Republic of China; 3 Neuro-Pathology Department, Beijing Neurosurgical Institute, No.6 Tiantan Xili, Dongcheng District, 100050 Beijing, People’s Republic of China

**Keywords:** Brain, Expression, Glioma, *p42.3*

## Abstract

**Background:**

Malignant gliomas are the most common form of primary malignant brain tumor. It has recently been suggested that genetic changes are involved in the progression of malignant gliomas. In previous studies, a novel gene, *p42.3*, was characterized as a tumor-specific gene that encodes a mitosis phase–dependent expression protein which is expressed in gastric cancer, but not in matched normal tissues.

**Methods:**

In a series of 200 human brain gliomas and 13 normal tissues, we performed RT-PCR and mRNA *in situ* hybridization for analysis of *p42.3* gene expression in gliomas, including astrocytoma (grade 2), oligoastrocytomas (grade 2), anaplastic oligoastrocytomas (grade 3), glioblastomas (grade 4) and normal tissues. Also, the mRNA expression was detected in gliomas by *in situ* hybridization. After producing polyclonal antibody to p42.3, we further tested p42.3 protein expression in astrocytomas and glioblastomas by immunohistochemistry and Western blot analysis.

**Results:**

Our results demonstrated that overexpression of the *p42.3* gene is detected in gliomas, but not in normal brain tissues. Importantly, *p42.3* mRNA expression is correlated with the pathological features of gliomas. In addition, p42.3 protein is expressed in both the cytoplasm and the nucleus in astrocytomas, whereas this protein appeared in the cytoplasm in glioblastomas.

**Conclusions:**

These results indicate that p42.3 might be involved in carcinogenesis as a potential molecular marker for malignant gliomas.

## Background

Gliomas are the most common human brain tumors; they account for more than 40% of central nervous system neoplasms [1]. These tumors are classified as astrocytomas, oligodendrogliomas (ODs) or oligoastrocytomas (OAs). With respect to their malignant progression, they are also categorized as grade 1, 2, 3 and 4 by the World Health Organization [2,3]. Grades 1 and 2 indicate benign tumors and grades 3 and 4 are malignant ones [4]. The most aggressive astrocytoma is referred to as glioblastoma multiforme (GBM) (grade 4) [5]. GBMs may develop *de novo* (primary GBMs) or by progression from low-grade or anaplastic astrocytomas (secondary GBMs) [6,7]. Molecular genetic study researchers have confirmed that genetic changes involved in the progression of malignant gliomas include loss of heterozygosity 10 (*LOH10*), phosphatase and tensin homolog (*PTEN*), tumor protein p53 (*TP53*), epidermal growth factor receptor (*EGFR*) and *P16*[8]. Despite advances in surgical and clinical neurooncology, the prognosis for patients with high-grade gliomas remains poor, owing to the insidious infiltration of these tumors into adjacent brain tissue [9,10]. Therefore, scientists need to perform more studies to clarify the detailed molecular mechanisms of gliomas and find biomarkers for early diagnosis or effective therapeutic targets in order to improve the prognosis for patients with malignant gliomas.

In previous studies, we confirmed that *p42.3* gene expression is cell cycle–dependent in gastric tumor cells and specifically expressed in gastric cancer, but not in normal mucosa [11]. In the present study, we were able to predict the *p42.3* gene expression profile in brain and liver tissues by digital Northern analysis. We confirmed gene expression in five kinds of tumor cell lines and fetal tissues, including those from the brain and liver. *p42.3* gene expression was studied in clinical specimens of gliomas and in normal human brain tissues for further analysis of the correlation between *p42.3* gene expression and malignant progression. Overall, our data support the hypothesis that the *p42.3* gene may be involved in tumorigenesis and might be a potential molecular marker for distinguishing different grades of malignant gliomas.

## Methods

### Ethics statement

All patients provided their written informed consent to participate in the study, and the clinical study was approved by the Institutional Review Board of Beijing Tiantan Hospital.

### Tissue samples

In this study, we used 162 paraffin-embedded samples, 51 nitrogen-frozen samples of human brain gliomas which that had been resected surgically and 13 normal tissues derived from traumatic decompression operations. Histopathologic analyses were independently performed by two pathologists.

### RT-PCR

Total RNA was extracted using TRIzol reagent. cDNA was synthesized from total RNA according to the manufacturer’s instruction manual (Promega, Madison, WI, USA). RT-PCR for analysis of the *p42.3* gene expression was performed using two pairs of primers (5′-TGG CAT CTT TAC TGG ACT GG-3′ and 5′-TGG CAC CTC GTG GAT AGA GC-3′; 5′-CCA GCG GCT ACC TGA CCT TC-3′and 5′-CAG CTC CTT CAT CTG CTT CAG-3′). The RT-PCR conditions were as follows: sufficient denaturing at 95°C for 50 seconds, denaturing at 94°C for 50 seconds, annealing at 55°C for 50 seconds, elongation at 72°C for 50 seconds (30 cycles) and a final cycle at 72°C for 10 minutes. β-actin served as the internal positive control. The RT-PCR products were examined by gel electrophoresis.

### mRNA *in situ* hybridization

The RT-PCR fragment of the *p42.3* gene was cloned into the pGEM-T easy vector system (Promega) that contained SP6 and T7 promoters. After sequencing, to confirm the orientation of the insert, the plasmids were linearized by either ApaI or SacI enzyme digestion. Digoxigenin (DIG)-labeled, anti-sense- or sense-strand RNA probes were prepared by *in vitro* transcription with T7 RNA polymerase and SP6 RNA polymerase according to the manufacturer’s instruction manual (Roche). A tissue array was incubated overnight at 43°C in prehybridization buffer containing 5 ng/μl of a sense or antisense DIG-labeled RNA probe. Detection of hybridization was undertaken using a DIG nucleic acid detection kit (Roche Applied Science, Indianapolis, IN, USA) according to the manufacturer’s instructions. Hybridization with sense-strand RNA probes was used as a negative control.

### Antibody generation and identification

Three peptides were selected on the basis of analysis using DNASTAR software (DNASTAR, Madison, WI, USA) and were synthesized by Shenggong Biological Company (Shenggong, China). We then immunized rabbits using these synthesized peptides to generate polyclonal antibodies. Preimmune and immune sera were harvested. The fragment of cDNA including CDS (coding sequence) was amplified by nested PCR and cloned into prokaryotic expression vector PET-28a. The p42.3 protein was expressed by *Escherichia coli* BL21 and purified by nickel nitrilotriacetic acid (Ni-NTA) agarose. The purified p42.3 protein was identified by matrix-assisted laser desorption/ionization time-of-flight mass spectrometry, and Western blot analysis was performed to identify the specificity of polyclonal antibodies. Polyclonal antibody to the p42.3 protein was isolated using purified protein and Sepharose 4B according to the manufacturer’s instructions (Borunlaite Science&Technology Company, Bejing, China).

### Western blot analysis

For protein analysis, tissues were lysed in protein lysis buffer. Protein (100 μg) was separated by 12% Tris-glycine polyacrylamide gel and transferred to a polyvinylidene fluoride membrane by electrophoretic blotting at 90 V for 2 hours. The membrane was blocked with 5% nonfat dry milk in 0.1% phosphate-buffered saline with Tween 20 (PBST) and was incubated with a 1:100 dilution of polyclonal antibody at 4°C overnight. The blot was then washed in PBST and incubated for 2 hours with horseradish peroxidase–conjugated secondary antibody goat anti-rabbit immunoglobulin G (IgG) (1:1,000) and washed in PBST. The membrane was then developed using an electrochemiluminescence Western blotting detection reagent system (Amersham, Little Chalfont, UK) according to the manufacturer’s instructions.

### Immunohistochemistry

Immunohistochemistry was performed as follows. Briefly, sections were deparaffinized by incubation at 70°C for 2 hours. Next, the slides were subjected to antigen retrieval by autoclave heating in an ethylenediaminetetraacetic acid buffer for 2.5 minutes and left to cool down to room temperature. After that, slides were then incubated in 3% H_2_O_2_ to block endogenous peroxidase after rehydration in ethanol gradient, and then they were incubated in 10% nonfat dry milk to reduce nonspecific binding. Afterward, the slides were sequentially incubated with purified anti-p42.3 antibody (1:50), biotinylated goat anti-mouse IgG and avidin-biotin complex were added for 30 minutes at room temperature and the slides were developed with diaminobenzidine (Dako, Carpinteria, CA, USA).

### Statistical methods

A *χ*^2^ test (SPSS 10.0 for Windows, Chicago, IL, USA) was performed to analyze the expression of p42.3 in grade 2 gliomas, and grade 4 GBMs were examined by RT-PCR (Table 1). Logistic regression analysis (SAS 8.01 software; SAS Institute, Cary, NC, USA) was carried out to assess the frequency of *p42.3* gene expression in gliomas identified by mRNA *in situ* hybridization (ISH) (Tables 2 and 3). Statistical significance was set at *P* < 0.05.

**Table 1:** **Frequency of *p42.3 *** **gene expression in gliomas identified by RT-PCR**^**a**^

**Histological specimens**	**Grade**	**Total cases, *N***	**p42.3 expression, *n***		**Percent positive**
			**Positive**	**Negative**	
Astrocytoma	2	11	1	10	9.1%
OA	2	10	1	9	10%
Anaplastic OA	3	6	0	6	0
GBM	4	11	5	6	45.5%
Normal tissue		9	0	9	0

^a^GBM, Glioblastoma multiforme; OA, Oligoastrocytoma.

**Table 2:** **Frequency of *p42.3 *** **gene expression in gliomas identified by mRNA *in situ *** **hybridization**^**a**^

**Tumor type (histological grade)**	**p42.3 expression (%)**	**Sex**	**Age <50 yr, *n***	**Age ≥50 yr, *n***	**Total ( *N *)**
Astrocytoma (grade 2)	Positive (15.9%)	Male	5	1	6
		Female	4	0	4
	Negative (84.1%)	Male	30	4	34
		Female	18	1	19
OD (grade 2)	Positive (9.52%)	Male	1	0	1
		Female	1	0	1
	Negative (80.48%)	Male	10	4	14
		Female	4	1	5
OA (grade 2)	Positive (12.5%)	Male	1	0	1
		Female	1	0	1
	Negative (87.5%)	Male	4	5	9
		Female	4	1	5
Anaplastic OA (grade 3)	Positive (30.0%)	Male	0	1	1
		Female	2	0	2
	Negative (70.0%)	Male	5	0	5
		Female	2	0	2
GBM (grade 4)	Positive (36.5%)	Male	9	5	14
		Female	4	1	5
	Negative (63.5%)	Male	13	10	23
		Female	6	4	10

^a^GBM, Glioblastoma multiforme; OA, Oligoastrocytoma; OD, Oligodendroglioma.

**Table 3:** **Analysis of correlation between *p42.3 *** **gene expression and pathological grade**^**a**^

**Characteristics**	**OR**	**95% CI**	***P***-**value**
Sex			0.4366
Female	Ref		
Male	1.384	0.610 to 3.137	
Age			0.5411
<50 yr	Ref		
≥50 yr	0.751	0.299 to 1.884	
Grade			0.0041*
2	Ref		
3 and 4	3.089	1.430 to 6.675	

^a^CI, Confidence interval; OR, Odds ratio; Ref, Reference value. *Statistically significant.

## Results

### Prediction of *p42.3* gene expression by digital Northern analysis

**Figure 1:**
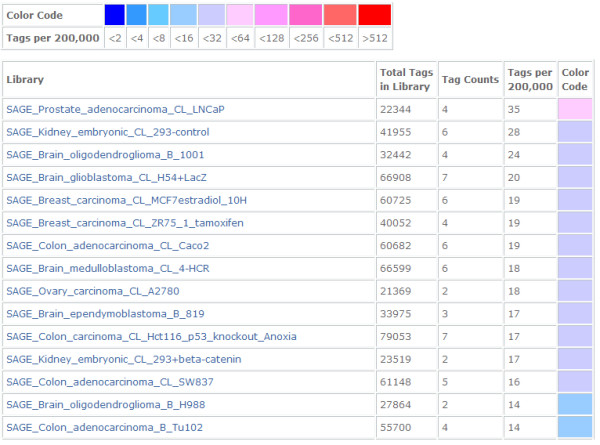
**Prediction of *p42.3 *gene expression in tumor cell lines and tissues.** Digital Northern analysis of the National Center for Biotechnology Information (NCBI) reference sequence showed that the *p42.3* gene is expressed in several kinds of cancer tissue, including prostate adenocarcinoma, brain glioblastoma multiforme, breast carcinoma and colon adenocarcinoma, as well as in embryonic kidney tissue.

We knew little about the novel gene *p42.3* after cloning its full-length cDNA. However, bioinformatics proved to be a very useful tool in helping us to find clues for a successive study; therefore, we predicted the expression profile by digital Northern analysis of the National Center for Biotechnology Information (NCBI) reference sequence. The results showed that the *p42.3* gene is highly expressed in several cancer tissues, such as prostate adenocarcinoma, brain GBM, breast carcinoma and colon adenocarcinoma, as well as in embryonic kidney tissue (Figure 1).

### mRNA expression of the *p42.3* gene is associated with degree of malignancy in gliomas

**Figure 2:**
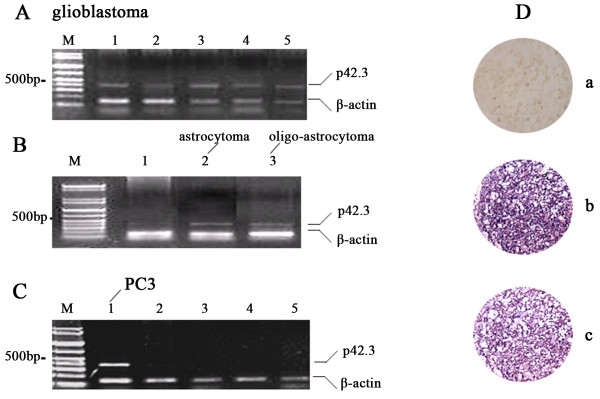
**Examination of *p42.3 *in brain gliomas. (A)** and **(B)***p42.3* expression was confirmed in five glioblastoma multiforme (GBM) samples, one astrocytoma sample and one oligoastrocytoma (OA) [it is correct here as written, “astrocytoma” and “oligoastrocytoma”.] sample by RT-PCR. **(C)***p42.3* was not expressed in normal brain tissue. The PC3 cell line served as the positive control [1]. **(D)** mRNA *in situ* hybridization analysis showed that *p42.3* was significantly overexpressed in anaplastic OA (b) and GBM (c), but not in normal brain tissue (a).

Digital Northern analysis showed that the *p42.3* gene is expressed in brain GBMs and confirmed that *p42.3* is expressed in human fetal brain tissue as well. Therefore, we performed RT-PCR to examine its expression in gliomas, including astrocytomas (grade 2), OAs (grade 2), anaplastic OAs (grade 3), GBMs (grade 4) and corresponding normal tissues. The results showed that the positive frequency of this gene expression was similar between astrocytomas (1 of 11, 9.1%) and OAs (1 of 10, 10%); however, we found that *p42.3* gene was expressed in 5 (45.5%) of 11 GBMs (Figure 2A and B) and was not expressed in 6 anaplastic OA samples*.* Moreover, this gene was undetectable in nine normal brain tissue samples (Figure 2C and Table 1). The prostate cell line PC3 served as a positive control. Using mRNA *in situ* hybridization with a variety of tissues, we examined *p42.3* gene expression in brain gliomas. Table 2 shows that the expression of this gene was positive in 10 (15.9%) of 63 astrocytomas, 2 (12.5%) of 16 OAs, 3 (30%) of 10 anaplastic OAs, 2 (9.52%) of 21 ODs and 19 (36.5%) of 52 GBMs. In contrast, the *p42.3* gene was not expressed in four normal brain tissues (Figure 2C and Table 2). More importantly, the statistical significance of expression between grade 2 and grade 4 gliomas was 0.038 (*P* < 0.05) (Table 1), and the expression of the *p42.3* gene was significantly different between low-grade (grade 2) gliomas and high-grade (grades 3 and 4) gliomas (*P* < 0.01), but there was no statistically significant difference associated with age or sex (Table 3).

### Polyclonal antibody binding of p42.3 protein

**Figure 3:**
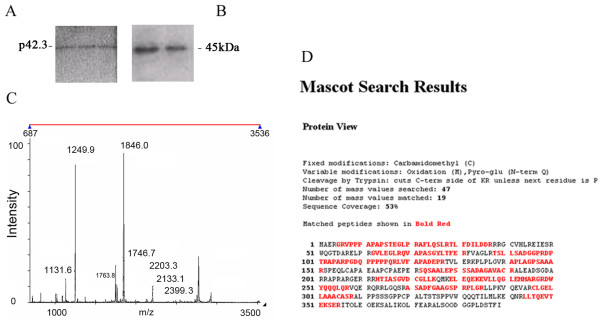
**Generation and identification of the p42.3 antibody. (A)** p42.3 was expressed in *Escherichia coli* and purified by nickel nitrilotriacetic acid agarose. **(B)** Western blot analysis showed that two sets of rabbit antiserum could specifically bind the purified protein of p42.3. **(C)** Mass spectrometry analysis confirmed that the sequence of purified protein corresponded to that of p42.3. **(D)** Mascot database (Matrix Science, Boston, MA, USA) search results.

We produced polyclonal antibody to the p42.3 protein to analyze the corresponding protein expression and generated six sets of rabbit antiserum against three synthetic peptide fragments of the p42.3 protein. The p42.3 protein was expressed in *E. coli* and purified by Ni-NTA agarose (Figure 3A). Western blot analysis showed that two of the six sets could specifically bind intact protein expressed in *E. coli* (Figure 3B).

### Frequent expression of p42.3 protein in glioblastoma multiforme

**Figure 4:**
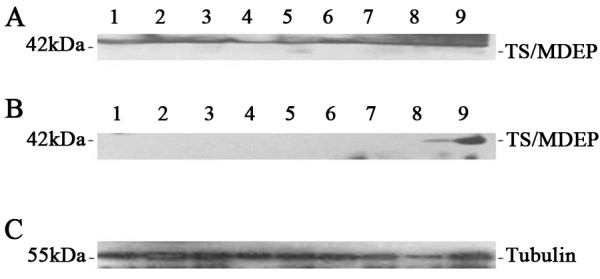
**Examination of p42.3 in glioma tissue samples by Western blotting. (A)** p42.3 protein was expressed in eight glioblastoma multiforme samples (grade 4, lanes 1 to 8) and one anaplastic oligoastrocytoma (OA) sample (grade III, lane 9). **(B)** p42.3 was detectable in two OA samples (grade 2, lanes 6 to 9; lanes 4 and 5 are anaplastic OA). The protein was not detected in three normal brain tissue samples (lanes 1 to 3). **(C)** Tubulin served as the internal control.

Western blot analysis was carried out to examine the p42.3 protein expression in gliomas and normal brain tissues. The glioma specimens included eight GBM samples (grade 4), four OA samples (grade 2), three anaplastic OA samples (grade 3), and three normal tissue samples. The results showed that p42.3 protein was frequently expressed in all eight GBM samples and detectable in one OA sample and one anaplastic OA sample. p42.3 protein was not expressed in the three normal tissue samples. β-actin served as an internal control (Figure 4).

### p42.3 protein is differentially located between astrocytomas and glioblastomas

**Figure 5:**
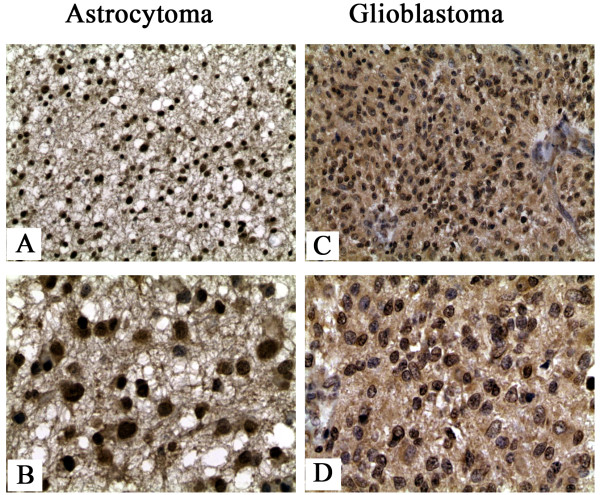
**p42.3 expression in astrocytomas and glioblastomas evaluated by immunohistochemistry.** p42.3 was expressed in both the cytoplasm (original magnification, ×200) **(A)** and the nucleus (original magnification, ×400) **(B)** in the astrocytoma samples (*n* = 10). p42.3 was expressed in the cytoplasm (original magnification, ×200) **(C)**, but not in the nucleus (original magnification, ×400) **(D)**, in the glioblastoma samples (*n* = 10). Logistic regression analysis (SAS 8.01 software) confirmed that the statistical difference between grade 2 and grades 3 and 4 tumors was significant (*P* < 0.01). No statistically significant difference associated with age or sex was found.

We further performed immunohistochemistry on ten astrocytoma samples and ten GBM samples to test p42.3 expression. Our results show that this protein was expressed in both astrocytomas (tumor grades 5 ≥ ++, 5+) and GBMs (tumor grades 4 ≥ ++, 6+). Moreover, the location of p42.3 expression is different in these two glioma types. p42.3 protein was tested in both the cytoplasm and nucleus in nine of ten astrocytoma samples (Figure 5A and B), especially the protein highly expressed in the nucleus. In contrast, this protein was strongly expressed in the cytoplasm in only nine of ten GBM samples, and there was little expression of p42.3 protein in the nucleus (Figure 5C and D).

## Discussion

In order to find out whether key genes are involved in both cell-cycle regulation and carcinogenesis, we screened out a cDNA fragment between the G_1_ and S phases of the cell cycle in a tumor cell line, BGC823, by mRNA differential display and then cloned the full-length cDNA, which was designated p42.3 [GenBank:DQ150361]. The *p42.3* gene is located on human chromosome 9q34.3 and is composed of five introns and six exons. It encodes a 389–amino acid (42.3 kDa) protein with functional motifs. In our previous study of gastric tumor cell lines and gastric cancer, we showed that the *p42.3* gene might be involved in cell-cycle regulation and might play a role in gastric cancer development [11].

A homolog of *p42.3* is present in the mouse, in which it is expressed in the early neuroectoderm of the embryo [12]. The high conservation of the *p42.3* gene in mammals also suggests that this gene might serve important roles in cell biology. Digital Northern analysis of the NCBI reference sequence showed that the *p42.3* gene is expressed in GBMs and other malignancies, which gave us important information about this novel gene. Our examination validated that the *p42.3* gene was indeed expressed in various cancer cell lines and fetal tissues, including the brain.

Gliomas are the most frequently encountered human brain tumors, and the prognosis for glioma patients worsens with increasing tumor grade. Patients with high-grade gliomas (for example, anaplastic astrocytomas and GBMs) have a median survival time of 9 months, and only 5% to 10% of patients can survive as long as 2 years [13,14]. Accumulation of multiple genetic abnormalities lead to human malignant gliomas. Inactivation of the p16-cdk4-pRb pathway has been implicated in the progression to grade 3 astrocytoma [15,16]. Secondary GBMs have a high incidence of *TP53* mutations (>65%), the majority of which are already present in low-grade or anaplastic astrocytomas [17]. *LOH10* is the most frequent genetic alteration in GBMs, occurring in 60% to 80% of cases [18-20]. Deletion of *PTEN*[21,22] and overexpression of *EGFR* have also frequently been found in GBMs [23,24]. These previous studies have elucidated the partial molecular mechanisms of malignant progression of gliomas, although the precise molecular events involved in the process are not yet known. Therefore, finding critical genes involved in the progression of malignancy might effectively confer an improved prognosis for patients with high-grade gliomas by application of targeted therapies.

The expression profile of the *p42.3* gene was the primary focus of our research in this study, in which we analyzed clinical tumor samples taken from patients with gliomas in China. The *p42.3* gene was confirmed to be expressed in these glioma samples, but not in normal human brain tissues, at both the mRNA and protein levels. More importantly, the percentage of *p42.3* mRNA expression increased in tandem with the progression of glioma malignancy, with statistically significant differences found between grade 2 (benign) tumors and grades 3 and 4 (malignant) tumors. This evidence is important evidence supporting the hypothesis that the *p42.3* gene might not only be involved in carcinogenesis but also play a role in the progression of glioma malignancy. Moreover, the different locations of p42.3 between astrocytoma and GBM cells suggests that this protein has different roles in high- and low-grade gliomas. Further study is needed to clarify these detailed functions in this process.

Interestingly, we found the same expression pattern in gliomas: The *p42.3* gene was expressed in glioma tumor tissue samples, but not in corresponding normal tissue, in our Chinese patient population. It is generally believed that a fetal protein, highly expressed at fetal stages and quickly shut down after birth, could recur in different tumor stages. Such molecules are likely to be a tumor marker and might act as an oncogene involved in carcinogenesis [25,26]. We found that the expression profile of the *p42.3* gene was in agreement with the criteria mentioned above.

## Conclusion

In our present study, we establish the expression pattern of a novel gene, *p42.3*, which suggests that this gene is associated with glioma malignancy. Moreover, we found that p42.3 mRNA expression was correlated with the degree of glioma malignancy. These results provide important clues to inform further studies of this novel gene and might be useful in diagnosing gliomas. The location of this protein may help investigators to discern high- and low-grade gliomas.

## Competing interests

The authors declare that they have no competing interests.

## Authors’ contributions

LZ and YL concepted and designed the study, TR, ZW, GJ, WW acquired clinical data, JZ and LZ performed the operation and collected the sample, ZW collected and transferred the samples, JW made pathological diagnosis, XX, WL and WW carried out the molecular genetic studies, JZ, LZ and GJ participated in the sequence alignment, TR, ZW and WW followed the patients. WW and XX carried out statistical analysis, WW drafted the manuscript. All authors critically revised the article. All authors read and approved the final manuscript.
